# Draft Genome Sequence of *Pseudomonas* sp. Strain LD120, Isolated from the Marine Alga *Saccharina latissima*

**DOI:** 10.1128/MRA.01305-19

**Published:** 2020-02-20

**Authors:** Clara Margot Heiman, Jutta Wiese, Peter Kupferschmied, Monika Maurhofer, Christoph Keel, Jordan Vacheron

**Affiliations:** a Department of Fundamental Microbiology, University of Lausanne, Lausanne, Switzerland; b Marine Ecology, Research Unit Marine Symbioses, GEOMAR Helmholtz Centre for Ocean Research Kiel, Kiel, Germany; c Plant Pathology, Institute of Integrative Biology, Swiss Federal Institute of Technology Zurich, Zurich, Switzerland; University of Maryland School of Medicine

## Abstract

We report the draft genome sequence of *Pseudomonas* sp. strain LD120, which was isolated from a brown macroalga in the Baltic Sea. The genome of this marine Pseudomonas protegens subgroup bacterium harbors biosynthetic gene clusters for toxic metabolites typically produced by members of this *Pseudomonas* subgroup, including 2,4-diacetylphloroglucinol, pyoluteorin, and rhizoxin analogs.

## ANNOUNCEMENT

*Pseudomonas* bacteria occur in many terrestrial and aquatic ecosystems, where they interact with various hosts, including plants, invertebrates, and humans (1–3). Pseudomonads can serve as a source for novel secondary metabolites, and some are used as bioremediation and biological control agents (4–6). The genus *Pseudomonas* is composed of different lineages, divided into groups and subgroups (7–9). The Pseudomonas protegens subgroup harbors mainly isolates from soil or roots that are active against plant pathogens and pest insects (5, 10, 11).

Here, we sequenced the genome of *Pseudomonas* sp. strain LD120 using PacBio technology. Strain LD120 was isolated from a blade of the brown macroalga Saccharina latissima, from the Baltic Sea (12). A 16S rRNA gene-based analysis placed LD120 in a close phylogenetic relationship with the P. protegens type strain CHA0 (13, 14). LD120 exhibits broad-spectrum antimicrobial activity, including activity against algal pathogens (12), which involves the toxic metabolites 2,4-diacetylphloroglucinol (DAPG) and pyoluteorin (14).

The MagAttract high-molecular-weight (HMW) DNA kit (Qiagen) was used to extract LD120 genomic DNA from 400 μl of an overnight nutrient yeast broth culture prepared from an individual colony derived from the original stock of the strain. Sequencing was performed by the Lausanne Genomic Technologies Facility. DNA samples were sheared in Covaris g-TUBEs to obtain fragments with a mean length of 10 kb. The sheared DNA was used to prepare a library with the PacBio SMRTbell template preparation kit v1.0. The resulting library underwent size selection on a BluePippin system (Sage Science, Inc.) for molecules larger than 7 kb, which excluded smaller plasmids. The library was multiplexed and sequenced using one single-molecule real-time (SMRT) cell and a Sequel system (movie length, 600 min). Genome assembly was performed using the RS_HGAP_Assembly.4 protocol in SMRT Link v6.0.

The resulting assembly generated six contigs with a maximum length and *N*_50_ value of 3,219,715 bp and 1,709,349 bp, respectively, providing a total genome length of 6,672,566 bp (G+C content, 61.61%; coverage, 136×). Annotation of the LD120 genome with the NCBI Prokaryotic Genome Annotation Pipeline (PGAP) identified 5,987 coding sequences. In addition, 68 tRNAs and 16 rRNAs were detected.

The average nucleotide identity (calculated based on BLAST+ results [15]) between the genomes of strain LD120 and the type strain CHA0 (16) was 87.32%, which identified LD120 as a member of the P. protegens subgroup. The LD120 genome harbors biosynthetic gene clusters for DAPG and pyoluteorin and also for rhizoxin analogs, which are produced by a subset of P. protegens strains (17–19). Unlike other P. protegens subgroup strains, LD120 does not harbor gene clusters for production of the antimicrobial pyrrolnitrin and the entomotoxin FitD (11, 20). This difference could be a result of coevolution with the algal host and points to genomic diversity within the P. protegens subgroup.

### Data availability.

This whole-genome shotgun project has been deposited in DDBJ/ENA/GenBank under accession number WVHL00000000. The version described in this paper is the first version, WVHL01000000. The sequences for the *de novo* assembly have been deposited in EMBL/GenBank under accession number ERR3588830.
